# Synthesis and NO_2_ Sensing Properties of In_2_O_3_ Micro-Flowers Composed of Nanorods

**DOI:** 10.3390/nano13162289

**Published:** 2023-08-09

**Authors:** Zhenyu Wang, Haizhen Ding, Xuefeng Liu, Jing Zhao

**Affiliations:** 1 School of Ocean Science and Technology, Dalian University of Technology, Panjin 124221, China; 2 School of Life Science and Medicine, Dalian University of Technology, Panjin 124221, China

**Keywords:** In_2_O_3_, micro-flowers, morphological evolution, NO_2_ sensor, solvothermal

## Abstract

Semiconductor oxide gas sensors have important applications in environmental protection, domestic health, and other fields. Research has shown that designing the morphology of sensitive materials can effectively improve the sensing characteristics of sensors. In this paper, by controlling the solvothermal reaction time, a unique hexagonal flower-like structure of In_2_O_3_ materials consisting of cuboid nanorods with a side length of 100–300 nm was prepared. The characterization results indicated that with the increase in reaction time, the materials exhibited significant morphological evolution. When the solvent heating time is 5 h, the flower-like structure is basically composed of hexagonal nanosheets with a thickness of several hundred nanometers and a side length of several micrometers. With the increase in reaction time, the apex angles of the nano sheets gradually become obtuse, and, finally, with the Ostwald ripening process, they become cuboid nanorods with side lengths of 100–300 nanometers, forming unique micro-flowers. Among them, the material prepared with a reaction time of 20 h has good sensing performance for NO_2_, exhibiting low operating temperature and detection limit, good selectivity, repeatability, and long-term stability, thus suggesting a good application prospect.

## 1. Introduction

In recent years, with the development of society, the issues of atmospheric pollution related to the emission of toxic and harmful gases as well as the safety hazards caused by the leakage of flammable and explosive gases have received widespread attention. As an important part of various gases studied previously, nitrogen dioxide (NO_2_) can lead to acid rain, photochemical smog and haze. Considering the great harm of NO_2_, the United States environmental protection agency (EPA) has recently established a new 1-h standard, namely 0.1 ppm, to provide requisite protection for public health, which is well below the previous value [1]. In view of these facts, it is particularly urgent to develop effective and reliable methods for detecting low concentrations of NO_2_.

Nowadays, gas sensors play a crucial role in detecting and quantifying combustible, explosive and harmful gases in many aspects of our daily life, typically in industrial processes, air quality monitoring, medical diagnosis, and public and domestic safety assurances. Therefore, there are many kinds of gas sensors that have been designed and used. Among them, the resistive gas sensor is the most attractive one due to its easy operation, convenient fabrication, low cost and small size [2,3,4]. Moreover, metal oxide has become a hot spot in the field of gas sensors since the first commercial gas sensor in the world adopted metal oxide as its sensitive material in 1968. Up to now, various metal oxides, including zinc oxide (ZnO) [5,6], tin dioxide (SnO_2_) [7,8], tungsten oxide (WO_3_) [9,10], titanium oxide (TiO_2_) [11,12], iron oxide (Fe_2_O_3_) [13,14], nickel oxide (NiO) [15,16], cobalt oxide (CO_3_O_4_) [17,18], copper oxide (CuO) [19,20,21] and indium oxide (In_2_O_3_) [22,23], have been investigated and applied to fabricating the gas sensors. Among them, due to the high conductivity and abundant defects, In_2_O_3_ has proven to be a good candidate for the detection of NO_2_. Generally, the working principle of In_2_O_3_ resistive gas sensors is associated with the resistance changes brought about by different gas atmospheres and working temperatures. Determined by the reductive or oxidative nature of the target gas, the gas molecules adsorbed on the metal oxide semiconductor surface will serve as an electron donor or acceptor via redox reactions. The gas–solid interface reactions alter the carrier concentration near the surface of the semiconductor, thus causing resistance changes in the sensors. Obviously, the morphology of the In_2_O_3_ material has a great influence on the performance of sensors. The sensitive material can obtain a larger specific surface area, less aggregation and more surface-active sites with the help of the proper design for its micro-morphology, which can significantly improve the sensing properties of the sensor. Hence, it is concluded to be very meaningful to explore the relationship between the morphology of In_2_O_3_ materials and the sensitive properties of sensors. Consequently, it is reported that numerous NO_2_ sensors have been fabricated with the In_2_O_3_ materials with various morphologies, including nanocubes [24], microcubes [25], thick films [26], nanotubes [27], nanorods [28] and spindles [29], etc. Thus, preparing multiple sets of In_2_O_3_ materials with different morphologies via a simple method and exploring the changes in their sensing characteristics with morphology evolution provide new inspiration for designing and fabricating NO_2_ sensors with tunable properties.

Based on the above considerations, this study prepared the unique In_2_O_3_ hierarchical micro-flowers composed of plates and nanorods by a facile solvothermal method and also characterized the effect of the solvothermal reaction time on the morphologies. In addition, this study fabricated and tested the sensors based on the materials as prepared in different reaction times. As shown in the results, the samples with a reaction time of 20 h exhibit high sensitivity, good selectivity, nice repeatability and outstanding long-term stability.

## 2. Materials and Methods

### 2.1. Synthesis of In_2_O_3_ Hierarchical Micro-Flowers

In a typical synthetic procedure, 0.3526 g (1.2 mmol) InCl_3_·4H_2_O (99.9% metals basis, Aladdin Reagent (Shanghai) Co., Ltd., Shanghai, China) was first dissolved in a 36 mL ethanol–water solvent (φ = 50%), and then 0.3 g SDS (C_12_H_25_SO_4_Na, Sodium dodecyl sulfate, Sinopharm Chemical Reagent Co., Ltd., Beijing, China) was dispersed in the above solvent by stirring and ultra-sonication. After that, 0.3 g urea (99.99% pure, Shanghai Macklin Biochemical Co., Ltd., Shanghai, China) was added and dissolved in the mixed solvent. Subsequently, the resulting mixture was transferred into a 50 mL Teflon liner to be sealed and heated in an oven at 120 °C for 20 h. After the mixture was naturally cooled to room temperature, its precipitate was washed with deionized water and ethanol and dried at 60 °C overnight. The dried precursor was then calcined in a muffle furnace at 500 °C with a residence time of 2 h to yield the In_2_O_3_ powder. Finally, the synthesized In_2_O_3_ samples were conducted at different solvothermal treatment times, in which the solvothermal treatment time was varied from 5 h to 25 h in time intervals of 5 h while keeping all other parameters fixed.

### 2.2. Physical Characterization Methods

The surface morphology and elemental composition details of In_2_O_3_ micro-flowers were determined by the field emission scanning electron microscope (SEM, Nova NanoSEM 450 model, FEI, Hillsboro, OH, USA) coupled with the EDAX detector. The structural characterization was analyzed by powder X-ray diffraction (XRD, D/MAX-2400 with Cu-Ka radiation of 1.54056 Å, Rigaku, Tokyo, Japan). Moreover, the specific surface areas of the sample were characterized by the Brunauer–Emmett–Teller (BET) method (Autosorb-iQ-C, Quantachrome Instruments, Boynton Beach, FL, USA).

### 2.3. Gas sensors Fabrication and Measurement

**Figure 1:**
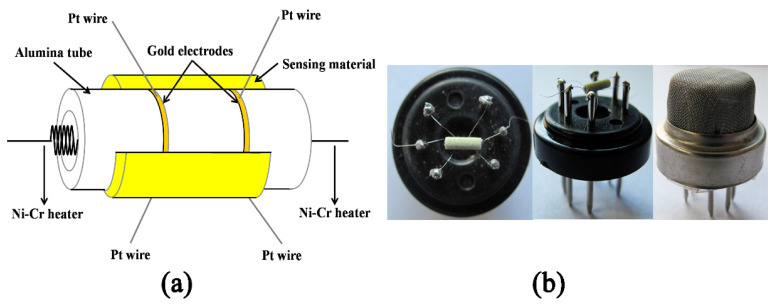
(**a**) The schematic diagram and (**b**) photographs of the gas sensor.

The gas sensing measurements were performed by depositing a thick film on an Al_2_O_3_ ceramic tube (4 mm in length, 1.2 mm in external diameter, and 0.8 mm in internal diameter, attached with a pair of gold electrodes). First, the proper amount of the gas sensing materials, namely as-prepared In_2_O_3_ micro-flowers, were mixed with deionized water for the paste. Then, the obtained paste was coated onto the ceramic tube and dried in air. After that, it was sintered at 500 °C to remove the organic residue and stabilize the signals. Meanwhile, a Ni–Cr alloy coil was inserted into the ceramic tube as a heater for ensuring that the sensor can work at an elevated temperature. The structure of the sensor is schematically shown in Figure 1. Furthermore, the changes in resistance during the adsorption and desorption of gas molecules on the surface of the sensing device are measured in air (R_a_) and in testing gas (R_g_). The electrical resistance was measured with a CGS-8 series Intelligent Test Meter (Beijing Elite Tech. Co., Ltd., Beijing, China), and the relative humidity of the atmosphere in the measurement is between 30 and 40%. The sensor response was calculated as S = R_g_/R_a_ for an oxidizing gas or S = R_a_/R_g_ for a reducing gas. In addition, the response and recovery times are defined as the time taken by the sensor to achieve 90% of the total resistance change in the case of adsorption and desorption, respectively. As mentioned above, the following was a typical testing procedure. First, the sensor was placed into the testing chamber full of air to get a steady state. And then, a calculated amount of target gas was injected into the chamber. With the help of the fans embedded in the testing system, the target gas was mixed with the air quickly and uniformly, thereby forming an atmosphere to be tested. When the resistance of the sensor reached a constant value, the chamber was opened to replace its internal gas with fresh air. Finally, the testing chamber was sealed and the sensor was kept inside it to achieve a complete recovery.

## 3. Results

### 3.1. Morphological and Structural Characteristics

**Figure 2:**
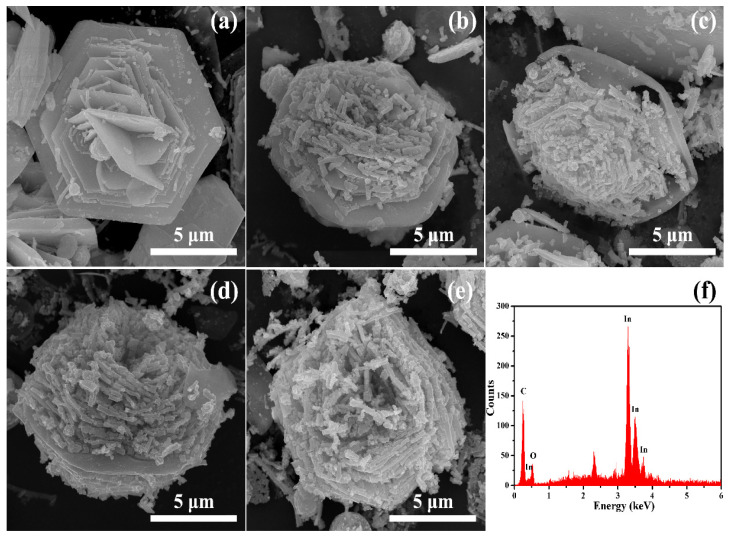
SEM images of In_2_O_3_ samples prepared at different reaction times: (**a**) 5 h, (**b**) 10 h, (**c**) 15 h, (**d**) 20 h and (**e**) 25 h. (**f**) EDS spectrum of In_2_O_3_ sample prepared at 20 h.

The surface morphologies of the as-prepared In_2_O_3_ samples were investigated by SEM images. In Figure 2, the images of In_2_O_3_ samples in different solvothermal reaction times clearly reveal that the solvothermal reaction time has great influence on the morphologies of the as-prepared In_2_O_3_ samples. According to the image of the In_2_O_3_ sample obtained with 5 h in Figure 2a, the morphology of an individual In_2_O_3_ microstructure indicates that the In_2_O_3_ sample is composed of regular hexagon In_2_O_3_ nanoplates with an edge length of about several microns and a thickness of about hundred nanometers. That is, in the microstructure, In_2_O_3_ has a unique flower-like morphology with a small amount of nanorods on its surface. Meanwhile, the In_2_O_3_ sample obtained with 10 h in Figure 2b has two obvious differences from that obtained with 5 h in Figure 2a: the apex angles of hexagon plates on the flower-like microstructures in Figure 2b are no longer sharp but slightly obtuse and the number of nanorods covering the “petals” in Figure 2b has a significant increase. Furthermore, as shown in Figure 2c, with further prolonging of the solvothermal reaction time to 15 h, the apex angles of the hexagon plates become obtuse and, meanwhile, there are some holes appearing on the hexagon plates. Furthermore, compared with Figure 2b, there is a large quantity of the nanorods on the flower-like microstructure in Figure 2c, which almost covers all “petals” of the microstructure. It should be noticed that the nanorods also grow roughly in accordance with the hexagonal trend. In Figure 2d, the number of the stacking layers of the nanorods has an increase with extending the solvothermal reaction time to 20 h. In addition, the hexagon in Figure 2d has not only smooth corners but also irregular edges, indicating that the hexagonal sheets are gradually degenerating with the increasing solvothermal reaction time. As shown in the side view in Figure 2d, numerous nanorods are stacked on both sides of the hexagon plate in the middle of the flower-like microstructure. As seen in the image of the In_2_O_3_ sample synthesized with the solvothermal reaction of 25 h in Figure 2e, there is no hexagon plate being observed any more due to the full coverage of nanorods and the flower-like microstructure, which has been transformed from a hexagonal structure to a pentagonal structure in some way. It can be seen that the synthesis conditions have a significant impact on the morphologies of the materials, which is consistent with the results in the literature [30]. In addition, research has shown that semiconductor nanorods have good redox properties, so we can expect the flower-like structures prepared in this study to have good gas sensing characteristics [31].

Moreover, the EDS spectrum was recorded to confirm the details of elements and composition for the proposed In_2_O_3_ flower-like microstructures. According to the EDS spectrum of the In_2_O_3_ sample with the solvothermal reaction time of 20 h in Figure 2f, it is confirmed that the sample exhibits the peaks corresponding to C, In and O elements, in which the C peak should correspond to those C elements in the substrate that are used to carry the material during the test.

**Figure 3:**
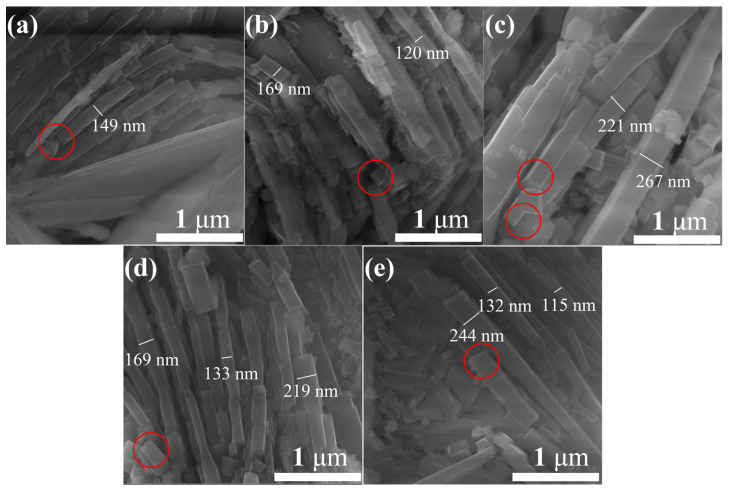
High magnification SEM images of In_2_O_3_ samples prepared at different reaction times: (**a**) 5 h, (**b**) 10 h, (**c**) 15 h, (**d**) 20 h and (**e**) 25 h. The red circles indicate the typical end faces of the nanorods.

In order to further study the micro morphology of the materials, they were characterized by SEM at a higher magnification. The results are shown in Figure 3. It can be seen that the nanorods that make up the micro flower are in the shape of cuboids (the red circles in the figure indicate the rectangular end faces of the nanorods), and the side length is about 100–300 nm. In Figure 3a, besides cuboid nanorods, nanosheets can also be seen, which is consistent with the results in Figure 2a. Based on the results of Figure 2 and Figure 3, it can be seen that the size of the nanorods does not change much with the increase in hydrothermal reaction time, but there is a significant difference in their quantity. Obviously, the material undergoes a process of first nucleation, then dissolution, and then nucleation. Therefore, it can be inferred that, with the increase in reaction time, the initially formed nanosheets gradually transformed into nanorods through the Ostwald ripening process.

**Figure 4:**
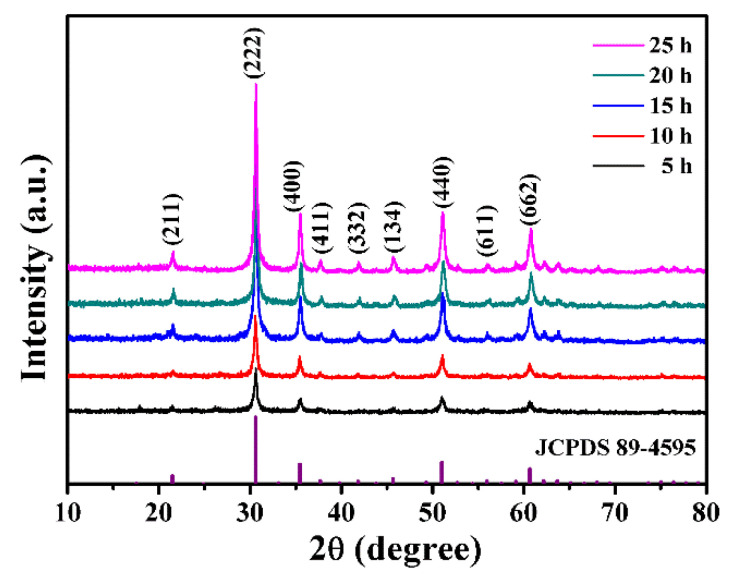
XRD patterns of In_2_O_3_ samples prepared at different solvothermal reaction times.

The phase purity and crystallographic structure of the In_2_O_3_ samples in different solvothermal reaction times were studied by XRD patterns. As shown in Figure 4, the XRD patterns of the as-synthesized In_2_O_3_ samples demonstrate (211), (222), (400), (411), (332), (134), (440), (611) and (662) reflection planes at 21.492, 30.578, 35.454, 37.683, 41.837, 45.678, 51.012, 55.970 and 60.653, respectively. All the diffraction peaks in these XRD patterns correspond to the cubic structure of In_2_O_3_, which are accorded well with those from the standard Joint Committee on Powder Diffraction Standards (JCPDS) card of In_2_O_3_, No. 89-4595. The observed patterns belong to the cubic crystal system with a space group Ia-3. The diffraction pattern of In_2_O_3_ with the characteristic diffraction peak at 2θ = 30.6° corresponds to the (222) plane, which confirms the formation of the cubic structure. In addition, there is no obvious diffraction peak being found besides that of In_2_O_3_, representing the good phase purity of the as-prepared products. With the increase of the solvothermal reaction time, the crystallinity of the sample becomes greater; coupled with that, the intensity of its diffraction peaks gradually increases despite it being very low with the reaction time of 5 h. Obviously, the diffraction peaks of the sample obtained with 25 h are much higher than that obtained with 5 h. The average crystallite sizes of In_2_O_3_ samples calculated by the Scherer formula are found to be 23.6, 24.1, 25.4, 27.2 and 28.1 nm for the solvothermal reaction time of 5 h, 10 h, 15 h, 20 h and 25 h, respectively. Thus, the calculated data indicates that crystallite size also increases with the prolongation of reaction time.

It is reported that the high value of the surface area is favorable for improving the gas sensing performance. In addition, the surface areas of the as-prepared In_2_O_3_ samples can be calculated by measuring nitrogen adsorption and desorption. With the help of the BET method, the BET surface areas are calculated as 10.476, 12.793, 14.469, 27.148 and 26.351 m^2^/g for In_2_O_3_ samples prepared with the solvothermal reaction times of 5 h, 10 h, 15 h, 20 h and 25 h, respectively. As shown in the results, the specific surface area of the sample increases gradually with the increase in solvothermal time and reaches its maximum at 20 h.

### 3.2. Gas-Sensing Properties

**Figure 5:**
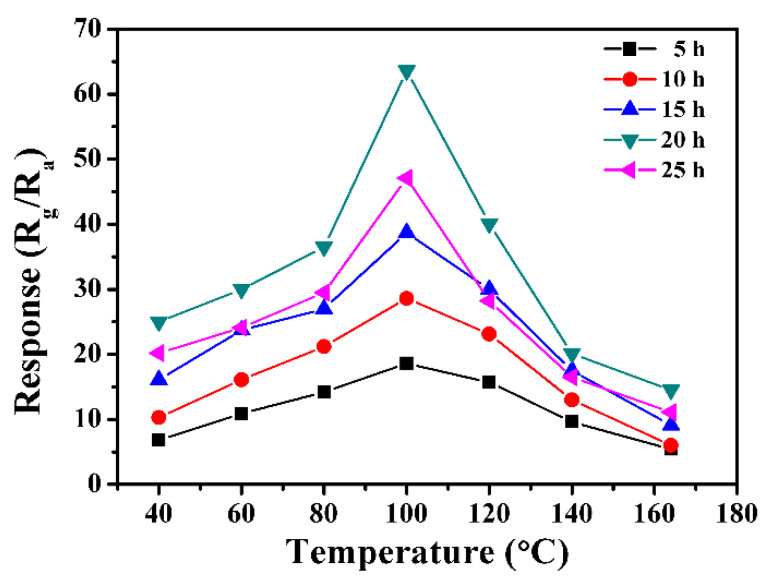
Response of sensors based on In_2_O_3_ samples prepared at different reaction times to 1 ppm NO_2_ as a function of the operating temperature.

Regulating the surface activity of sensitive materials and the adsorption/desorption process of gas molecules, operating temperature not only plays an important role in determining the specific interaction between NO_2_ and the sensing material but also has a great effect on the final sensing response. In order to determine an optimum operating temperature, the response of the In_2_O_3_-based sensor samples to 1 ppm NO_2_ was examined as a function of operating temperature. As shown in Figure 5, the five samples, respectively, with the reaction times of 5 h, 10 h, 15 h, 20 h and 25 h all have the largest response to 1 ppm of NO_2_ at the operating temperature of 100 °C. Initially, all the responses continuously increase with the increase of the operating temperature until reaching the maximum value at 100 °C, and then decrease with the further increase of the operating temperature. The characteristic volcano curve can be interpreted by the adsorption and reaction processes occurring on the sensing layer surface, which are greatly affected by the operating temperature. It is also known that it is necessary for the chemisorption and reaction on the surface to overcome the activation-energy barrier. At temperatures below 100 °C, the adsorbed NO_2_ molecules are not activated to react well with the surface adsorbed oxygen species due to lacking enough energy, thereby leading to a low response. With the increase in working temperature, the response value increases synchronously due to the increase in the surface reaction rate. However, when the temperatures are above 100 °C, the increase in the surface reaction rate cannot compensate for the negative effects posed by the difficulty of gas molecular adsorption and the low depth of diffusion, which leads to the decrease of sensor response value with the increase in temperature. Only at 100 °C, there is an optimum balance between the reaction and adsorption for the NO_2_ molecules. Therefore, as the optimal operating temperature, 100 °C is adopted in the following measurement. In addition, since the sample obtained with 20 h has the largest response at 100 °C compared with the other four samples, 20 h is defined as the most appropriate solvothermal reaction time in preparing the In_2_O_3_ sample to detect the toxic NO_2_ gas.

**Figure 6:**
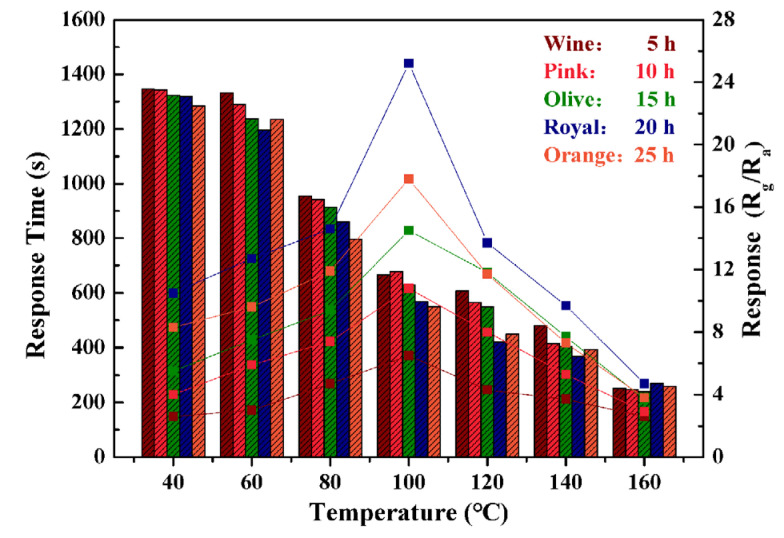
The response and response time of materials synthesized with different solvent thermal reaction durations to 500 ppb NO_2_ at different operating temperatures.

Figure 6 shows the response and response time of materials synthesized with different solvent thermal reaction durations to 500 ppb NO_2_ at different operating temperatures, which can help us further study the working temperature’s effect on the sensing properties of the sensors. It can be seen that when the operating temperature is only 40 °C, the response time of the sensor is as long as 1300 s, and the response value to 500 ppb NO_2_ does not exceed 10. As the working temperature increases, the response value of the sensor gradually increases and the response time also decreases. Specifically, the response time at 60 °C has a small change compared to 40 °C, and from 80 °C onwards, the response time is significantly shortened. In addition, at 100 °C, the response value significantly increased, reaching its maximum value, with the material obtained with a reaction time of 20 h having the highest response. As the working temperature further increases, the response time also decreases, but the response value also shows a significant decrease. Based on the comprehensive analysis of response value and response time results, 100 °C is still the optimal operating temperature for the sensor, which is consistent with the result we determined previously.

**Figure 7:**
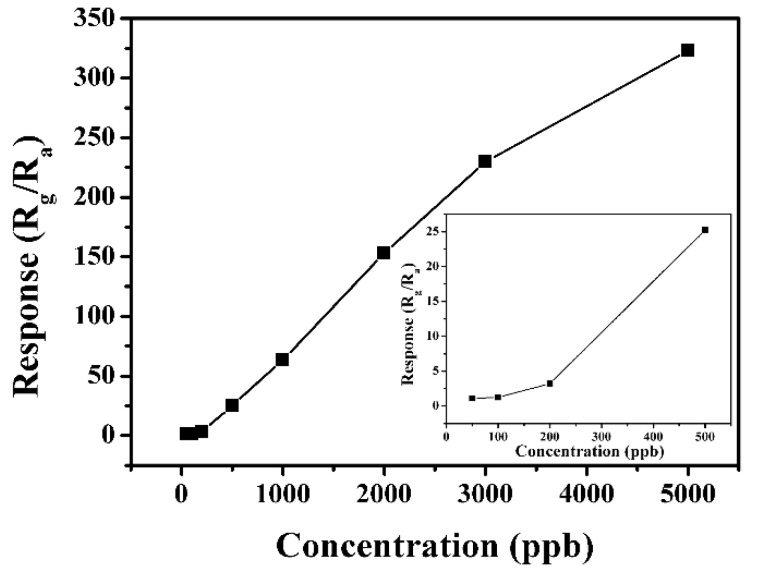
The response of the sensor based on the In_2_O_3_ sample prepared with 20 h to different concentrations of NO_2_ at 100 °C.

As depicted in Figure 7, the response, as a function of NO_2_ concentration for the sensor fabricated with the In_2_O_3_ sample obtained with 20 h, is investigated to further understand the sensor. It can be observed that the response values of the sensor increase when increasing the NO_2_ concentrations from 50 ppb to 5 ppm. The response of the sensor based on In_2_O_3_ micro-flowers has a rapid increase when the NO_2_ concentration is below 2 ppm, but its increasing trend slows down when the NO_2_ concentration is above 2 ppm, indicating that the response is gradually saturating. The responses of the sensor are 1.1, 1.2, 3.2, 25.2, 63.6, 153.0, 229.7 and 322.9, corresponding to the NO_2_ with concentrations of 50 ppb, 100 ppb, 200 ppb, 500 ppb, 1 ppm, 2 ppm, 3 ppm and 5 ppm, respectively. The inset of Figure 7 shows the gas response of the sensor to the NO_2_ with a lower concentration clearly. It is worthy to notice that the response can also be measured even though the sensor based on the as-prepared In_2_O_3_ micro-flowers was exposed to only 50 ppb NO_2_.

**Figure 8:**
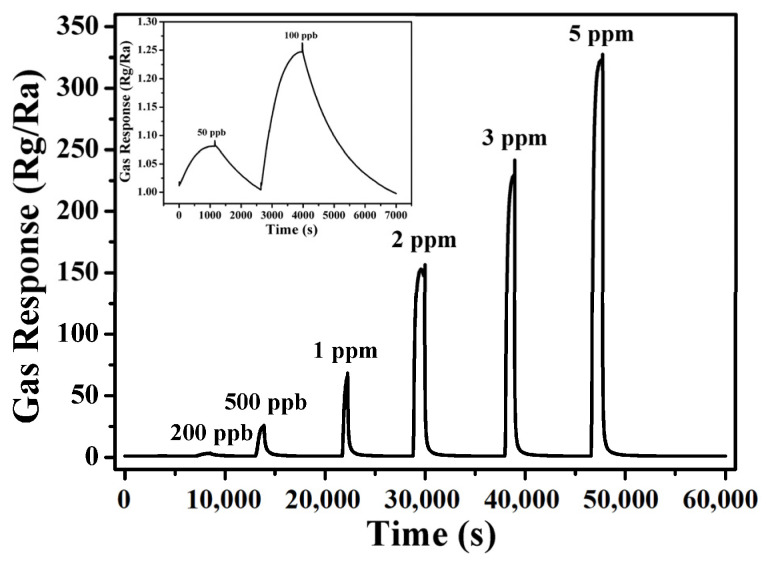
Dynamic response and recovery curve of the sensor fabricated with the In_2_O_3_ sample prepared with 20 h to different concentrations of NO_2_ at 100 °C.

Figure 8 shows the dynamic response and recovery curves of the gas sensor that was exposed to the NO_2_ of different concentrations ranging from 50 ppb to 5 ppm at the optimal operating temperature (100 °C). As shown in Figure 8, although the sensor can recover completely after detecting any concentration of NO_2_ gas, it needs to take a long time in its response recovery process when the concentration of NO_2_ gas is low. As shown in the inset figure, the sensor still has an obvious response to NO_2_ with a concentration of as low as 50 ppb, which indicates that it has a potential application in detecting the NO_2_ with low concentrations.

**Figure 9:**
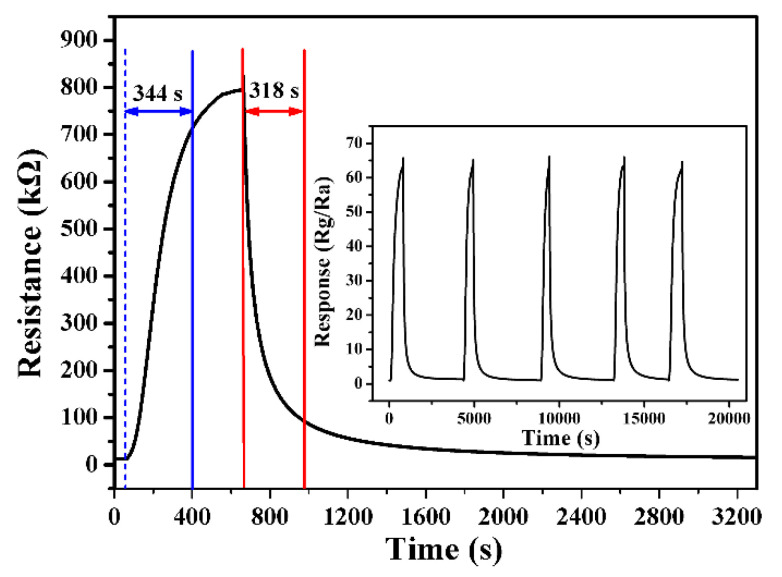
The response of sensors based on the In_2_O_3_ sample prepared with 20 h to 1 ppm NO_2_ at 100 °C. The inset displays five periods of the response curve at 100 °C.

Figure 9 presents a typical response profile of the sensor based on the In_2_O_3_ micro-flowers to NO_2_ at 100 °C. When the sensor was exposed to 1 ppm NO_2_, its resistance increased gradually from 12.5 to 795 kΩ, and its calculated response and response time were 63.6 and 344 s, respectively. When the resistance of the sensor reached a constant value, the gas sensor was placed in fresh air for a complete recovery, with the corresponding recovery time of 318 s. As is known, reproducibility and stability are critical properties of a gas sensor. The five reversible cycles of the response–recovery curves to 1 ppm NO_2_ in the inset of Figure 9 demonstrate that the sensor has excellent stability and repeatability.

**Figure 10:**
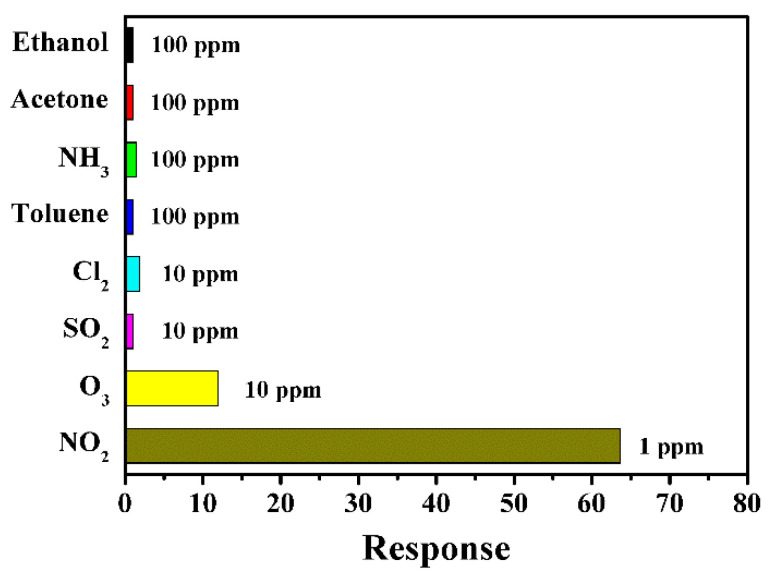
The response of the sensor based on the In_2_O_3_ sample prepared with 20 h to various gases at 100 °C.

In practical application, selectivity and long-term stability are significant parameters for gas sensors. Figure 10 shows the responses of the sensor to various kinds of testing gases, including ethanol, acetone, NH_3_, toluene, Cl_2_, SO_2_, O_3_ and NO_2_, at the operating temperature of 100 °C. Obviously, the sensor fabricated with In_2_O_3_ micro-flowers has an excellent response to NO_2_, but has weak responses to other kinds of gas, indicating that the sensor has a quite prominent selectivity towards NO_2_.

**Figure 11:**
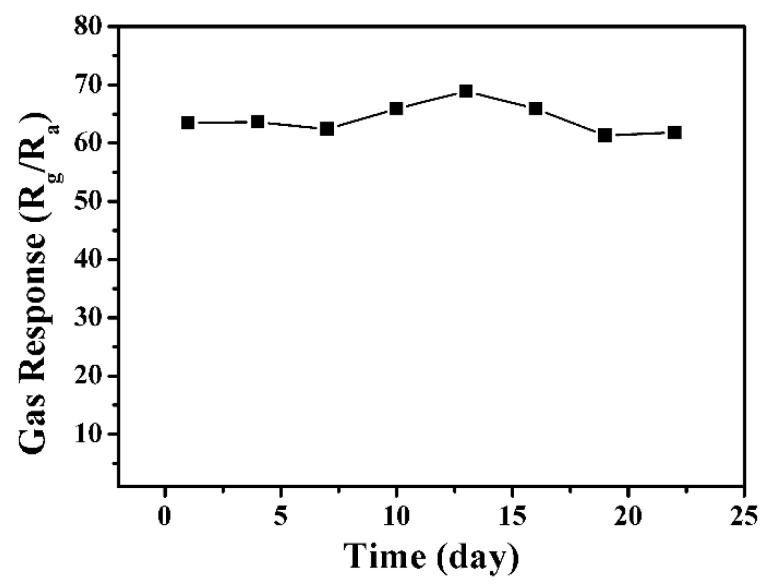
The long-term stability of the sensor based on the In_2_O_3_ sample prepared with 20 h at 100 °C.

Figure 11 depicts the responses of the sensor to 1 ppm NO_2_ at 100 °C, in which the data were measured every three days during a three-week period. As shown in the monitored results, the responses only have very slight changes after running for 3 weeks, which reveals that the sensor has good long-term stability for NO_2_ detection.

Table 1 summarizes and lists the NO_2_ gas-sensing performances of different N-type semiconductor-based gas sensors. Compared with the works in the previous literature, the as-prepared sensor in this work has obvious advantages with regards to the working temperature and the response value, which are conducive to its potential applications in NO_2_ detection.

### 3.3. Gas Sensing Mechanism

The gas-sensing properties of the In_2_O_3_ sensors are evaluated by measuring the changes in electrical resistance brought by the chemical reactions between the gas molecules and the sensing materials. The interaction of gas with the surface of In_2_O_3_ micro-flowers is basically related to the process of adsorption, reaction and desorption [32,34,44,45].

Initially, oxygen molecules (O_2_ (gas)) in the air are adsorbed physically (O_2_ (ad)) on the surface of micro-flowers (Equation (1)). With the increase in temperature, oxygen molecules are chemically adsorbed by In_2_O_3_ and are then ionized to be adsorbed oxygen species, such as O_2_^−^, O^−^ and O^2−^ (Equations (2) and (3)). During this process, electrons are trapped from the conduction band of In_2_O_3_, and a depletion layer is formed on the surface of the In_2_O_3_ grain, resulting in an increase in the resistance for In_2_O_3_ sensors.

(1) $$O_{2}\;(\mathrm{gas})\;\to \;O_{2}\;(\mathrm{ad})$$

(2) $${O_{2}\;(\mathrm{ad})+e}^{-}\;\to {\;O}_{2}^{-}\;(\mathrm{ad})\;(T\;<\;100\;{}^{\circ}C)$$

(3) $${O_{2}\;(\mathrm{ad})+2\;e}^{-}\;\to {\;2O}^{-}\;(\mathrm{ad})\;(100\;{}^{\circ}C\;<\;T\;<\;300\;{}^{\circ}C)$$

(4) $${O_{2}\;(\mathrm{ad})+2\;e}^{-}\;\to {\;O}^{2-}\;(\mathrm{ad})\;(T\;>\;300\;{}^{\circ}C)\;$$

When In_2_O_3_ is exposed to the NO_2_ at 100 °C, NO_2_ gas molecules can not only be adsorbed on the surface of In_2_O_3_ and capture electrons directly from the conduction band (Equations (5) and (6)), but also react with chemisorbed oxygen species to obtain electrons (Equations (6) and (7)), which is conducive to greatly increasing the depletion layer thickness of the material’s surface as well as significantly improving the resistance of the sensor.

(5) $${\mathrm{NO}}_{2}\;(\mathrm{gas})\;\to {\;\mathrm{NO}}_{2}\;(\mathrm{ad})$$

(6) $${\mathrm{NO}}_{2}{\;(\mathrm{ad})+e}^{-}\;↔{\;\mathrm{NO}}_{2}^{-}\;(\mathrm{ad})$$

(7) $${\mathrm{NO}}_{2}{\;(\mathrm{ad})+O}^{-}{\;(\mathrm{ad})+2e}^{-}\;↔{\;\mathrm{NO}}_{2}^{-}{\;(\mathrm{ad})+O}^{2-}\;(\mathrm{ad})$$

(8) $${\mathrm{NO}}_{2}{\;(\mathrm{ad})+O}_{2\;}^{-}{(\mathrm{ad})+2e}^{-}\;↔{\;\mathrm{NO}}_{2}^{-}{\;(\mathrm{ad})+2O}^{-}\;(\mathrm{ad})$$

The good sensing properties of the sensor can be attributed to the unique hexagonal flower-like structure of the In_2_O_3_ materials. On the one hand, as a unit for forming the sensing film, an individual flower-like structure was composed of many cuboid nanorods, which provided a lot of exposed active sites, grain boundaries and diffusion pathways to enhance the reaction between NO_2_ molecules and In_2_O_3_ micro-flower. On the other hand, when considered as a whole, the unique structure of In_2_O_3_ sensing materials can effectively prevent the aggregations among adjacent micro-flowers after being coated on the surface of a ceramic tube to form sensing layers. Loose and porous sensing layers are conducive to the diffusion of the target gas from the surface to the interior, which not only improves the utilization rate of the sensitive layer, thereby improving the response of the sensor, but also accelerates the adsorption and desorption processes of gas molecules on the material surface, thereby shortening the response recovery time of the sensor [29]. Therefore, even when exposed to lower concentrations of NO_2_, the sensor exhibits good sensing performance.

## 4. Conclusions

In summary, In_2_O_3_ micro-flowers were synthesized by facile solvothermal and subsequent heat treatment methods. Time-dependent experiments revealed that as the solvothermal reaction time increases, the constituent units of micro-flowers gradually changed from hexagonal nanosheets with a thickness of several hundred nanometers and side lengths of several micrometers to more and more cuboid nanorods with side lengths of 100–300 nanometers. Obviously, the solvothermal time has a significant impact on the morphology of the material, and the material exhibits significant morphological evolution as the solvothermal reaction time changes. This phenomenon is inferred to be caused by the Ostwald ripening process. Gas sensors fabricated with the In_2_O_3_ micro-flowers obtained with 20 h exhibited the best sensing properties to NO_2_, so 20 h is believed to be the most suitable reaction time length. Its optimal working temperature was as low as 100 °C, and its response to 1 ppm NO_2_ was 63.6, in which corresponding response and recovery time were, respectively, 344 and 318 s. Notably, the sensor showed an obvious response to NO_2_ with concentrations as low as 50 ppb. Additionally, the tests of selectivity, repeatability and long-term stability also prove the excellent characteristics of the sensor. Therefore, it is believed that 20 h is the most suitable reaction time.

## Figures and Tables

**Table 1:** NO_2_ sensing properties of N-type semiconductor-based gas sensors evaluated in this work and those reported in the literature.

Sensing Materials	Method	T (°C)	Resp. (Rg/Ra)	Con. (ppm)	Ref.
In_2_O_3_ Microcubes	Hydrothermal	100	17.3	1	[25]
Porous In_2_O_3_ Nanosheets	Solvothermal	250	164	50	[32]
In_2_O_3_ Octahedron	Thermal decomposition	200	43.52	30	[33]
In_2_O_3_ Thick Films	Spray pyrolysis	150	33.45	100	[26]
Micro Flower	Hydrothermal	125	19.6	5	[34]
rGO-In_2_O_3_ Hybrid	Solvothermal	150	22.3	0.5	[35]
Pt-In_2_O_3_	Hydrothermal	165	7.5	5	[36]
Co_3_O_4_/In_2_O_3_	Hydrothermal	150	27.9	10	[37]
In_2_O_3_ Nanocubes	Solvothermal	300	55.6	100	[24]
Pt-WO_3_ Films	Glancing angle deposition	150	11.24	1	[38]
Pd-SnO_2_ Nanowires	Vapor–Liquid–Solid	200	21.87	10	[31]
Pd-TiO_2_ Nanofiber mats	Electrospinning	180	38	2.1	[39]
Au-Co_3_O_4_ Nanoparticles	Reduction	136	136	0.1	[40]
Au-SnO_2_/NiO Thin films	Sputtering	200	180	5	[41]
Fe_2_O_3_ Nanoparticles	RF magnetron sputtering	150	1.69	100	[42]
α-MnO_2_ cube	Hydrothermal	150	1.33	100	[43]
In_2_O_3_ Micro-flower	Solvothermal	100	63.6	1	This work

## Data Availability

The data that support the findings of this study are available from the corresponding authors upon reasonable request.
